# Supplementary material for: Non-human primate model of long-COVID identifies immune associates of hyperglycemia
Source: Nat Commun. 2024 Aug 20;15:6664. doi: 10.1038/s41467-024-50339-4 (PMC11335872; doi:10.1038/s41467-024-50339-4)
Supplement: Supplementary file 1 — Supplementary Information [file 41467_2024_50339_MOESM1_ESM.docx]

**Supplementary information**

**Non-Human Primate Model of Long-COVID Identifies Immune Associates of Hyperglycemia**

Clovis S. Palmer^1,2,9^, Chrysostomos Perdios^1,2,9^, Mohamed Abdel-Mohsen^3^, Joseph Mudd^1,2^, Prasun K. Datta^1,2^, Nicholas J. Maness^1,2^, Gabrielle Lehmicke^1^, Nadia Golden^1^, Linh Hellmers^1^, Carol Coyne^1^, Kristyn Moore Green^1^, Cecily Midkiff^1^, Kelsey Williams^1^, Rafael Tiburcio^4^, Marissa Fahlberg^1^, Kyndal Boykin^1^, Carys Kenway^1^, Kasi Russell-Lodrigue^1,5^, Angela Birnbaum^1,8^, Rudolf Bohm^6^, Robert Blair^1,7^, Jason P. Dufour^1,5^, Tracy Fischer^1,2^, Ahmad A. Saied^1,7^, Jay Rappaport^1,2^

^1^Tulane National Primate Research Center, Covington, LA, USA; ^2^Department of Microbiology and Immunology, Tulane University School of Medicine, New Orleans, LA, USA; ^3^The Wistar Institute, Philadelphia, PA, USA; ^4^Division of Experimental Medicine, Department of Medicine, University of California, San Francisco. ^5^Department of Medicine, Tulane University School of Medicine, New Orleans, LA, USA. ^6^Oregon National Primate Research Center, Oregon Health and Science University, Beaverton, OR, USA. ^7^Department of Pathology and Laboratory Medicine, Tulane University School of Medicine, New Orleans, LA, USA.

^8^Deceased: Angela Birnbaum. ^9^These authors contributed equally: Clovis S. Palmer, Chrysostomos Perdios.

Corresponding Authors:

Clovis S Palmer, cpalmer3@tulane.edu

Jay Rappaport, jrappaport@tulane.edu

**Key words:** SARS-CoV-2, COVID-19, Diabetes, hyperglycemia, Long-Covid, PASC, Vaccine, metabolism, immunometabolism.

**Supplementary figures**

**
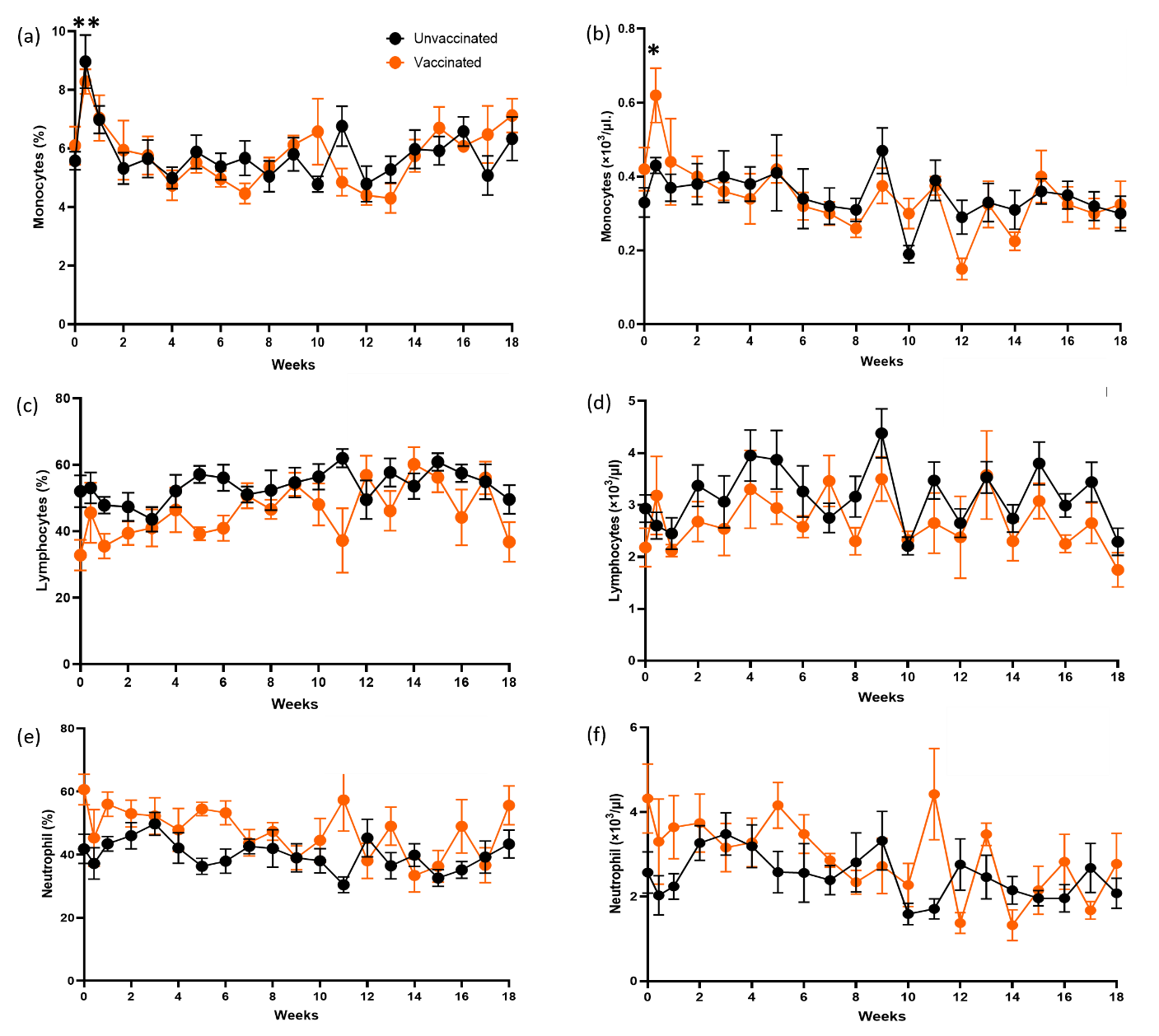
**

**Figure S1. Analysis of major immune cells in blood of infected AGMs over time**. (a-b) Changes in the percentage and absolute counts of monocytes, (c-d) lymphocytes, and (e-f) neutrophils in SARS-CoV-2 infected vaccinated or unvaccinated AGMs. Error bars represent SEM**.***p<0.01; **p<0.001. Statistical comparison between groups was done using the two-sided Mann Whitney U test. Source data are provided as a Source Data file.


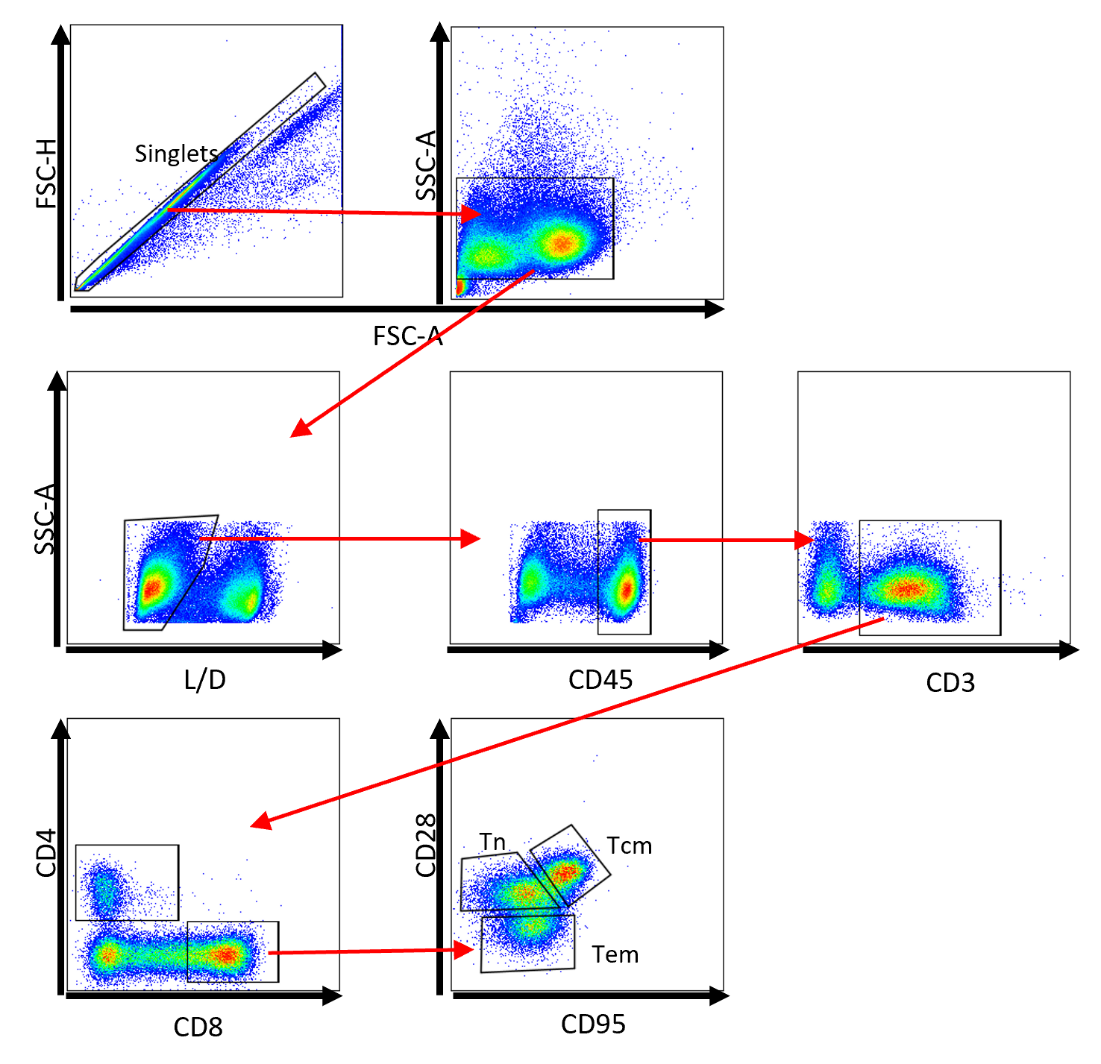


**Figure S2**. **Gating strategy for Spectre and T cell in vitro responsiveness analysis.** Red arrows show the gating steps. Tn = T naive; Tcm = T central memory; Tem = T effector memory. Total memory was defined as Tcm + Tem.


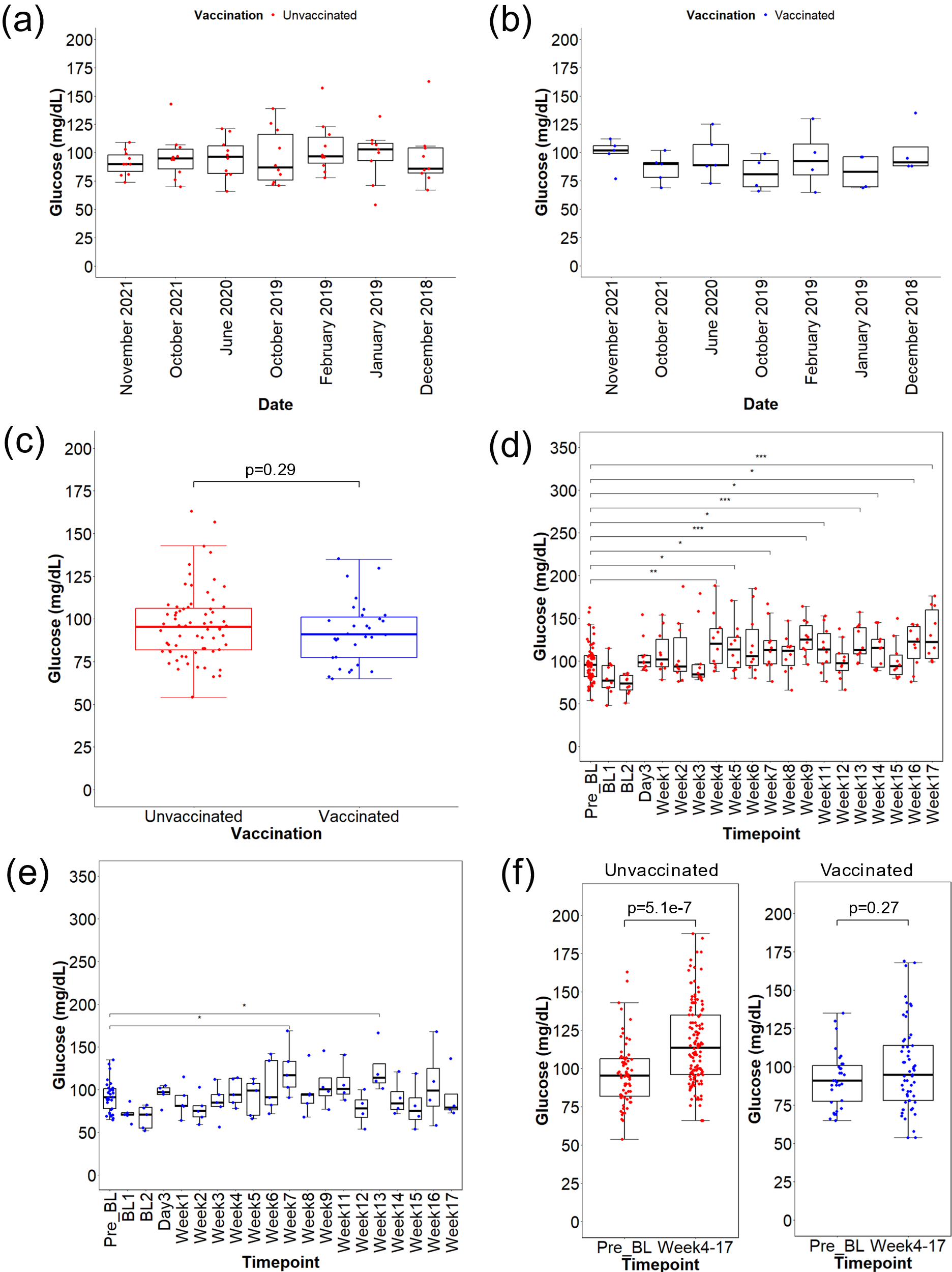


**Figure S3. Glucose levels during pre-baseline and study timeline.** (a) Non-fasted glucose levels over a three-year period before the baseline (pre-BL) in the future appointed vaccinated and (b) unvaccinated animals. (c) Future vaccinated and unvaccinated animals had non significantly differential glucose levels between them before the study (pre-BL). (d) The cumulative post-infection glucose levels were significantly higher in 9 of the 16 weeks examined in the unvaccinated group (e) and in 2 of the 16 weeks examined in the vaccinated group, when compared to pre-baseline measurements. (f) Unvaccinated and vaccinated blood glucose readings from all the pre-BL timepoints vs week 4 to week 17 p.i. in both groups. Statistical comparison between groups was done using the two-sided Mann Whitney U test. Whiskers represent 1.5x the interquartile range. *p<0.05; **p<0.01; ***p<0.001; ****p<0.0001. Source data are provided as a Source Data file.


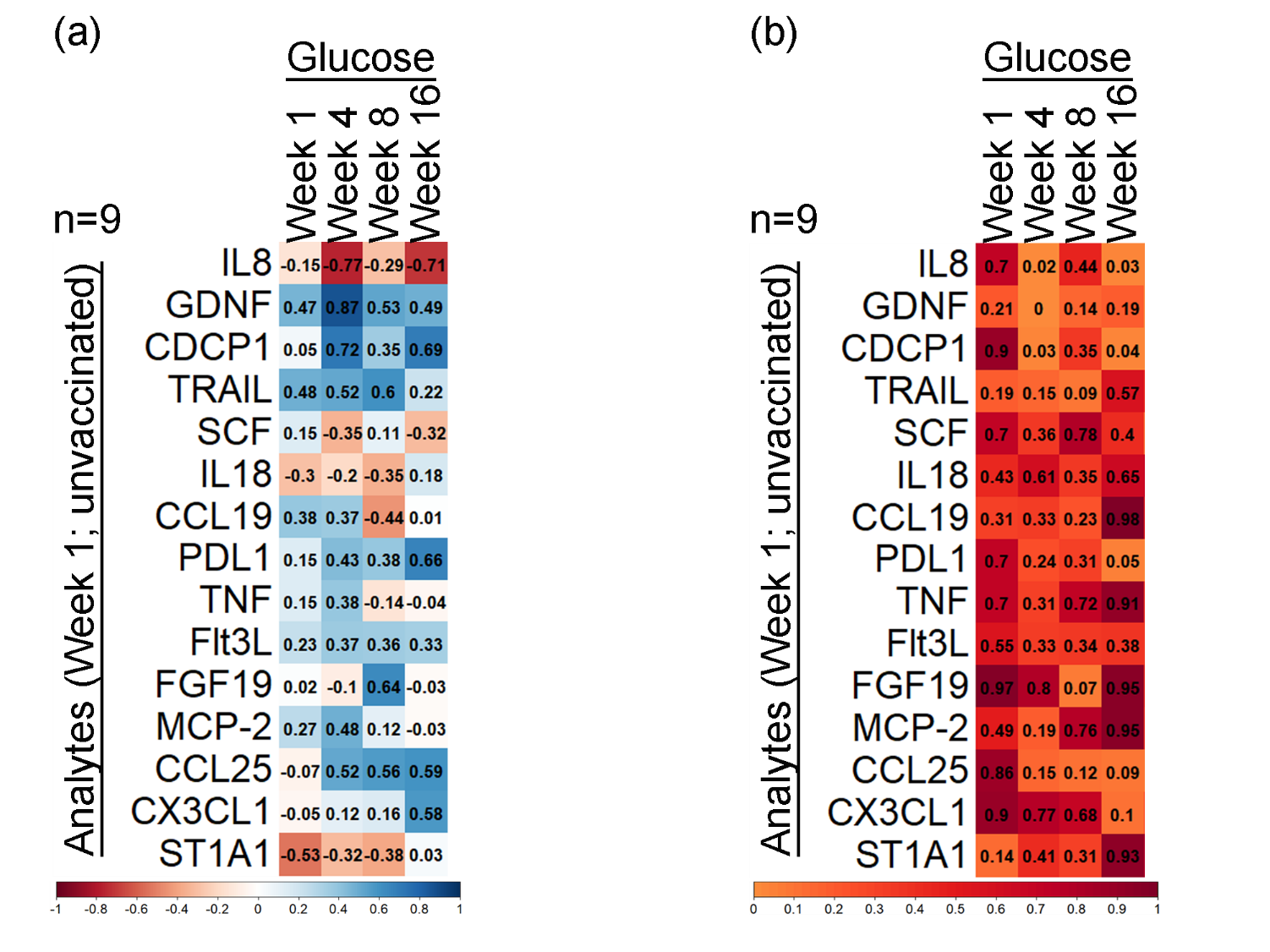


**Figure S4. Correlation analysis between blood glucose and analytes regulated at week 1 in unvaccinated animals**. (a) Correlation matrix depicting the correlation r values between SARS-CoV-2-modulated analytes at week 1, and plasma glucose concentrations at specific timepoints. (b) Correlation matrix depicting the correlation raw p values between SARS-CoV-2-modulated analytes at week 1, and plasma glucose concentrations at specific timepoints. Two-sided Spearman’s rank correlation was used for statistical analysis. Source data are provided as a Source Data file.


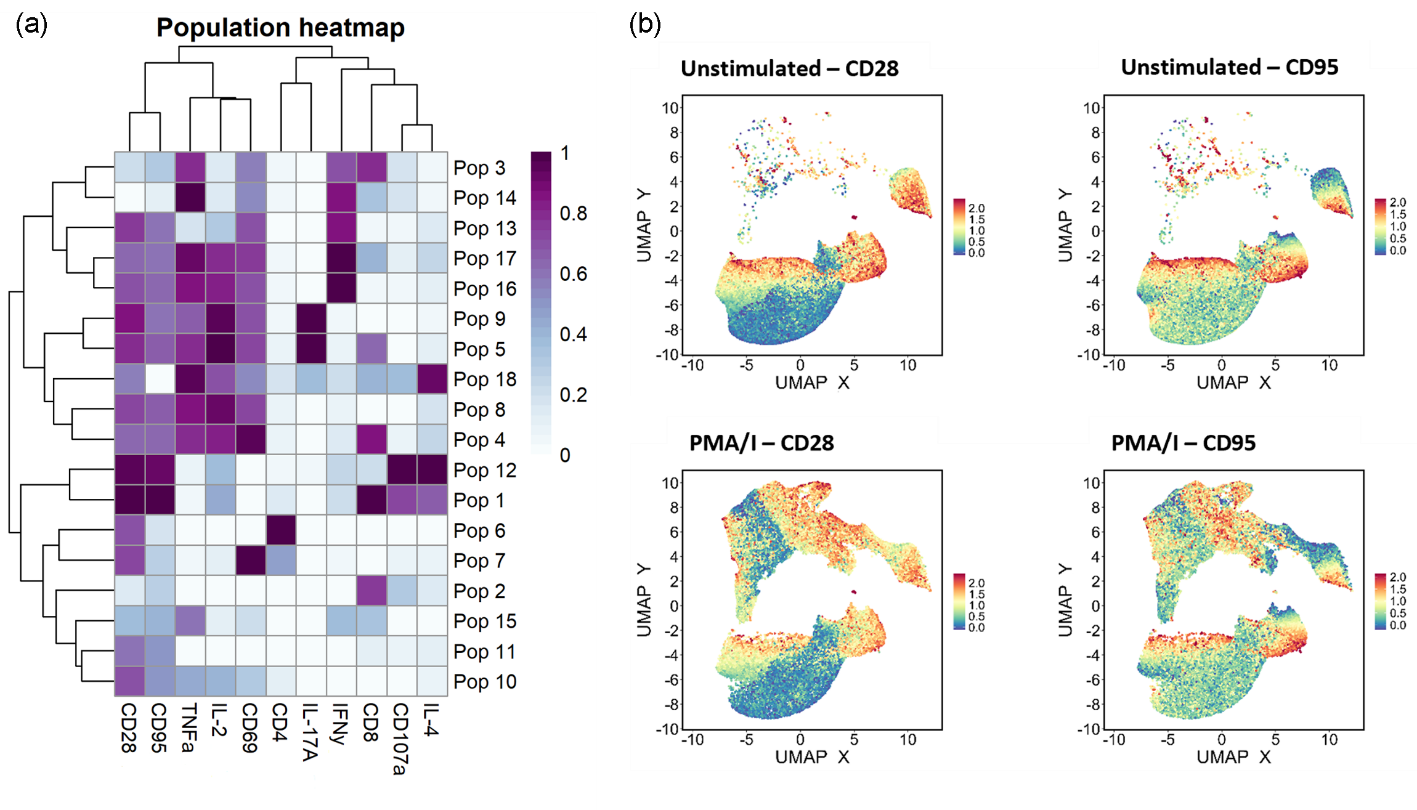


**Figure S5. Heatmap from Spectre analysis show populations in untreated PBMCs, and PMA/I treated cells**. (a) The heatmap used to analyze and identify the unstimulated and PMA/I cell populations presented in Fig. 5. CD4+ T cells are also defined as CD3+CD8- cells due to down regulation of CD4 upon PMA/I activation. (b) Expression patterns of maturation markers CD28 and CD95 in unstimulated and PMA/I treated cells.


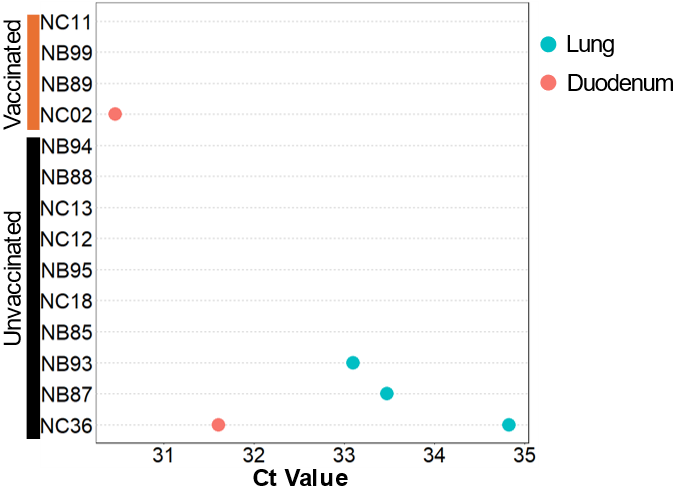


**Figure S6. Expression of SARS-CoV-2 genomic N in tissues**. qPCR analysis on tissues collected from 14 animals (vaccinated, n=4; unvaccinated, n=10) at 18 weeks p.i. Genomic N signal was found in the duodenum of 2 animals and in the lung of 3 animals.


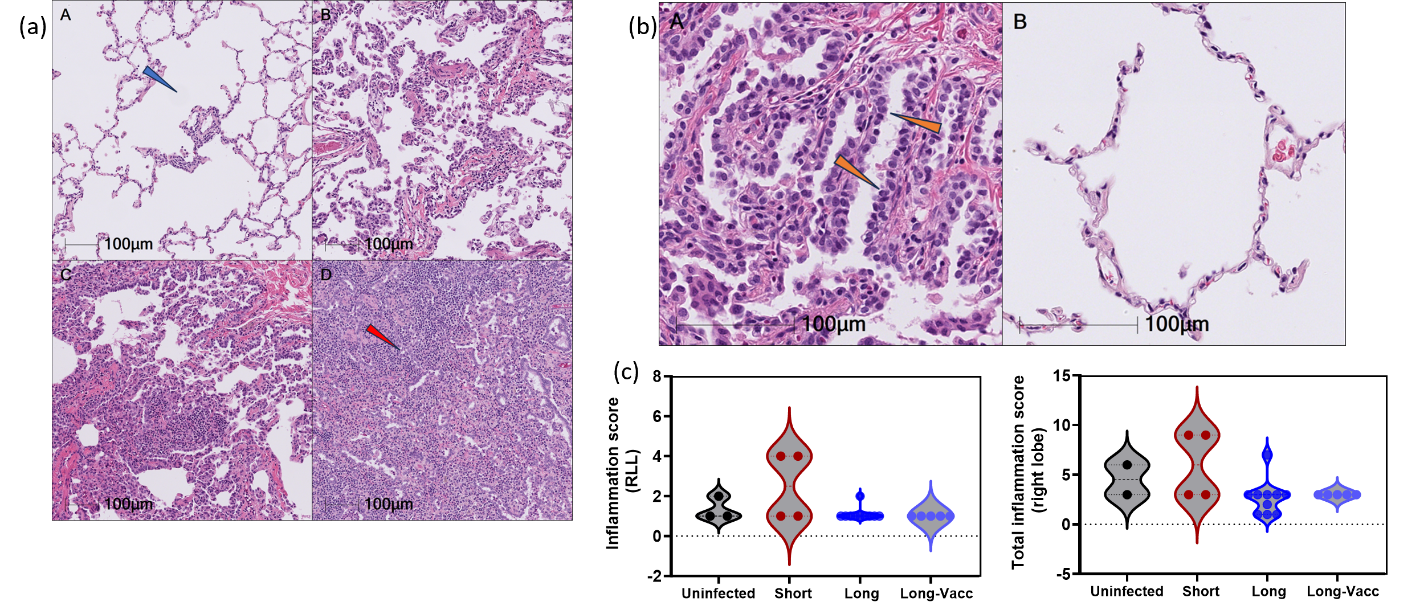


**Figure S7.** **Histopathology of left anterior lungs showing representation of scoring scale used for analysis.** Lung histopathology, hematoxylin & eosin stain. The histological changes in the lung were scored on the scale of 0 to 4. The changes evaluated for scoring include inflammation, pneumocyte type II hyperplasia, and fibrous connective tissue formation. (a) shows representative pictures of the histological changes and the scores. Panel A Score 1; minimal changes. Blue arrow indicates expanded alveolar spaces. Panel B. Score 2; mild changes. Panel C. Score 3; moderate changes. Panel D. Score 4; marked changes. Red arrow indicates diminished alveolar spaces due to infiltrating inflammatory cells. (b) Panel A shows a representative picture of pneumocyte type II hyperplasia from an animal infected with SARS-CoV-2. Orange arrows show marked pneumocyte type II hyperplasia. Panel B shows relatively normal alveolar septa with no pneumocyte type II hyperplasia for comparison. (c) Inflammation score of the right lower lungs (RLL, left), and composite inflammation score of the RLL (right). The scores consider the numbers of noticeable inflammatory cells present, pneumocyte type II hyperplasia, and the degree of fibrous connective tissue formation. Source data are provided as a Source Data file.


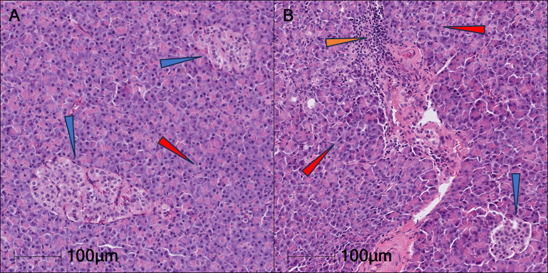


**Figure S8. Representative H&E images of pancreas from SARS-CoV-2 infected AGMs.**  (A) is given an inflammation Score 0, no visible inflammation. The Islets of Langerhans (blue arrows), and exocrine pancreas (red arrow) are within normal limits. (B) is given a Score 1, minimal inflammation. The blue arrow shows an islet of Langerhans with no significant histological changes. Inflammation (orange arrow) is often within the exocrine pancreas, and periductular tissue.

**Table S1. Significantly regulated plasma analytes between baseline and week 1 in SARS-CoV-2 infected AGMs.**

| **Regulated between BL and week 1** | | |
| --- | --- | --- |
| **Analytes** | **p-value*** | **BH (FDR)*** |
| CCL25 | 0.00391 | 0.04766 |
| CDCP1 | 0.00391 | 0.04766 |
| Flt3L | 0.00391 | 0.04766 |
| MCP-2 (CCL8) | 0.00391 | 0.04766 |
| SCF | 0.00391 | 0.04766 |
| TRAIL | 0.00781 | 0.07943 |
| CCL19 | 0.01171 | 0.08936 |
| IL-18 | 0.01171 | 0.08936 |
| FGF-19 | 0.01953 | 0.10831 |
| GDNF | 0.01953 | 0.10831 |
| IL-8 | 0.01953 | 0.10831 |
| CX3CL1 | 0.02734 | 0.11120 |
| PD-L1 | 0.02734 | 0.11120 |
| ST1A1 | 0.02734 | 0.11120 |
| TNF | 0.02734 | 0.11120 |

*Statistical analysis, Wilcoxon signed-rank test; two -sided; with BH correction.

**Table S2. Functionally enriched networks based on significantly regulated plasma analytes between baseline and week 1 in SARS-CoV-2 infected AGMs.**

| **Functional enrichment in network** | **BH (FDR)** |
| --- | --- |
| **Biological process (Gene Ontology)** | |
| Regulation of leukocyte migration | 1.38E-06 |
| Positive regulation of leukocyte migration | 5.55E-06 |
| Regulation of cell population proliferation | 6.39E-06 |
| **Molecular function (Gene Ontology)** | |
| Receptor ligand activity | 9.73E-14 |
| Cytokine activity | 1.66E-13 |
| Cytokine receptor binding | 5.26E-11 |
| **Local network cluster (STRING)** | |
| Chemokine receptors bind chemokines, and macrophage proliferation | 7.74E-06 |
| Regulation of dendritic cell dendrite assembly, and I-selectin | 0.00015 |
| Chemokine receptors bind chemokines | 0.00015 |
| **KEGG pathways** | |
| Viral protein interaction with cytokine and cytokine receptor | 1.01E-12 |
| Cytokine-cytokine receptor interaction | 2.08E-09 |
| Chemokine signaling pathway | 2.60E-05 |
| **WikiPathways** | |
| Allograft rejection | 0.00042 |
| IL-18 signaling pathway | 0.00048 |
| COVID-19 adverse outcome pathway | 0.0167 |
| **Tissue expression (TISSUES)** | |
| Polymorphonuclear leukocyte | 0.0063 |
| Intestinal epithelial cell | 0.0063 |
| THP-1 cell | 0.0063 |

**Table S3. Two-sided Spearman correlation analysis between blood glucose (week 0, 1, 4, 12) and plasma analytes (Olink data) in unvaccinated SARS-CoV2-infected AGMs.**

| **Correlation across weeks: analytes vs glucose** | | | |
| --- | --- | --- | --- |
| **Analytes** | **r** | **p-value** | **BH (FDR)** |
| CCL25 | 0.574 | 0.0003 | 0.0044 |
| GDNF | 0.556 | 0.0004 | 0.0066 |
| ADA | 0.445 | 0.007 | 0.0490 |
| ST1A1 | 0.432 | 0.009 | 0.0592 |
| CXCL9 | 0.431 | 0.009 | 0.0596 |
| IL-10RB | 0.412 | 0.013 | 0.0764 |
| IL-8 | -0.410 | 0.014 | 0.0828 |
| FGF-19 | 0.388 | 0.019 | 0.1024 |
| CDCP1 | 0.354 | 0.034 | 0.1446 |

**Table S4. Antibodies used in cytometry experiments.**

| **Antigen** | **Flurochrome** | **Manufacturer** | **Catalog #** | **Optimal Concentration (based on Titration Data)** | **Titrated Working Test Volume** |
| --- | --- | --- | --- | --- | --- |
| Live/Dead | Zombie Aqua | BD | 564406 | 1X | 6µl |
| CD45 | FITC | BD | 557803 | 1X | 20µL |
| CD3 | BV650 | BD | 563916 | 2X | 10µl |
| CD4 | BV786 | BD | 563914 | AGM - 0.5X ; RM - 2X | AGM - 2.5µL ; RM - 10µL |
| CD8 | BUV737 | BD | 749367 | 1X | 5µL |
| CD28 | BV605 | BioLegend | 302967 | 2X | 10µL |
| CD95 (Fas) | BV711 | BioLegend | 305643 | 1X | 5µL |
| CD69 | PE-CF594 | BioLegend | 310941 | 1X | 5µL |
| IL-2 | BB700 | BioLegend | 500321 | AGM - 2X ; RM - 1X | AGM - 10µL ; RM - 5µL |
| TNFα | APC | BioLegend | 502913 | 1X | 5µL |
| IFNy | PE-Cy7 | BioLegend | 502527 | 2X | 10µl |
| IL-4 | BV421 | BioLegend | 500825 | 2X | 10µl |
| IL-17A | PE | BioLegend | 512305 | 1X | 5µL |
| CD107a | BUV395 | BD | 565113 | 1X | 5µL |
