# Supplementary material for: Non-human primate model of long-COVID identifies immune associates of hyperglycemia
Source: Nat Commun. 2024 Aug 20;15:6664. doi: 10.1038/s41467-024-50339-4 (PMC11335872; doi:10.1038/s41467-024-50339-4)
Supplement: Supplementary file 3 — Reporting Summary [file 41467_2024_50339_MOESM3_ESM.pdf]

nature portfolio

Corresponding author(s): Clovis Palmer and Jay Rappaport

Last updated by author(s): 2024/02/23

Reporting Summary

Nature Portfolio wishes to improve the reproducibility of the work that we publish. This form provides structure for consistency and transparency in reporting. For further information on Nature Portfolio policies, see our Editorial Policies and the Editorial Policy Checklist.

Statistics

For all statistical analyses, confirm that the following items are present in the figure legend, table legend, main text, or Methods section.

n/a	Confirmed	
☐	☒	The exact sample size (n) for each experimental group/condition, given as a discrete number and unit of measurement
☒	☐	A statement on whether measurements were taken from distinct samples or whether the same sample was measured repeatedly
☐	☒	The statistical test(s) used AND whether they are one- or two-sided<br>*Only common tests should be described solely by name; describe more complex techniques in the Methods section.*
☐	☒	A description of all covariates tested
☒	☐	A description of any assumptions or corrections, such as tests of normality and adjustment for multiple comparisons
☐	☒	A full description of the statistical parameters including central tendency (e.g. means) or other basic estimates (e.g. regression coefficient) AND variation (e.g. standard deviation) or associated estimates of uncertainty (e.g. confidence intervals)
☐	☒	For null hypothesis testing, the test statistic (e.g. F, t, r) with confidence intervals, effect sizes, degrees of freedom and P value noted<br>*Give P values as exact values whenever suitable.*
☒	☐	For Bayesian analysis, information on the choice of priors and Markov chain Monte Carlo settings
☒	☐	For hierarchical and complex designs, identification of the appropriate level for tests and full reporting of outcomes
☐	☒	Estimates of effect sizes (e.g. Cohen's d, Pearson's r), indicating how they were calculated

Our web collection on statistics for biologists contains articles on many of the points above.

Software and code

Policy information about availability of computer code

Data collection	Plasma analyte OLINK data were obtained from the OLINK Analysis Services Lab in Waltham (MA, USA) or at the High Containment Research Performance Core (TNPRC). Flow cytometry data were acquired BD FACSymphony™. For RNA scope imaging and quantitation, brightfield images were acquired using the Axio Observer 7 (Zeiss), equipped with ZEN blue edition software (v3.6.096.08000).
Data analysis	R was used to perform statistical analysis, principal components analysis, and ggplot2 (v 3.3.3) was used to create principal component plot, heat maps, and correlation matrices. GraphPad prism (version 9.0) was also used for statistical analysis. FlowJo software (v 10.8.1) and Spectre (v 1.1.0) R package were used for flow cytometry analysis.<br><br>For RNA scope analysis, images were subjected to brightness and contrast enhancement in Photoshop (Adobe, v24.4.0) applied to the entire image. Slides were scanned with the Axio Scan.Z1 digital slide scanner (Zeiss). Scanned files were analyzed with HALO (Indica Labs, v3.4.2986.151) algorithm ISH (v4.1.3). Protein-protein interactions were analyzed using (Search Tool for the Retrieval of Interacting Genes/ Proteins (v 11.5), PANTHER (Protein Analysis Through Evolutionary Relationships; v 17.0; http://www.pantherdb.org/) classification system, and BioGRID (v 4.4; https://thebiogrid.org/). Figure 1c-e R code.txt: https://github.com/chrysperdios/Non-Human-Primate-Model-of-Long-COVID-Identifies-Immune-Associates-of-Hyperglycemia/tree/main

For manuscripts utilizing custom algorithms or software that are central to the research but not yet described in published literature, software must be made available to editors and reviewers. We strongly encourage code deposition in a community repository (e.g. GitHub). See the Nature Portfolio guidelines for submitting code & software for further information.

Data

Policy information about availability of data

All manuscripts must include a data availability statement. This statement should provide the following information, where applicable:

- Accession codes, unique identifiers, or web links for publicly available datasets
- A description of any restrictions on data availability
- For clinical datasets or third party data, please ensure that the statement adheres to our policy

The authors declare that data supporting the results and conclusions of this study are available within the paper and its supplementary information. Additional data that support the findings of this study are available from the corresponding authors upon reasonable request. The OLINK data generated in this study have been deposited in the Figshare database license CC BY 4.0, available at https://figshare.com/s/1259afc65d4fcd09b14c

Research involving human participants, their data, or biological material

Policy information about studies with human participants or human data. See also policy information about sex, gender (identity/presentation), and sexual orientation and race, ethnicity and racism.

Reporting on sex and gender	N/A
Reporting on race, ethnicity, or other socially relevant groupings	N/A
Population characteristics	N/A
Recruitment	N/A
Ethics oversight	N/A

Note that full information on the approval of the study protocol must also be provided in the manuscript.

Field-specific reporting

Please select the one below that is the best fit for your research. If you are not sure, read the appropriate sections before making your selection.

☒ Life sciences ☐ Behavioural & social sciences ☐ Ecological, evolutionary & environmental sciences

For a reference copy of the document with all sections, see nature.com/documents/nr-reporting-summary-flat.pdf

Life sciences study design

All studies must disclose on these points even when the disclosure is negative.

Sample size	This long COVID animal model is new with no preexisting longer-term data in non-human primates at the start of the study. Our main goal is to assess the long-term metabolic, inflammatory and clinical consequences of COVID-19 including neurological pathology. In our previous short-term study using SARS-CoV-2 infected African green monkeys we were able to detect significant differences in neuronal pathology and cell death at a magnitude of p≤0.005 using 4 biologically independent uninfected control group and n=8 biologically independent SARS-CoV-2 infected group. Here we use an additional animal in the vaccinated group (n=5), and 2 additional (n=8) to account for any unforeseen death before the end of the study period.<br><br>Reference: Rutkai, I. et al. Neuropathology and virus in brain of SARS-CoV-2 infected non-human primates. Nat Commun 13, 1745, doi:10.1038/s41467-022-29440-z (2022).
Data exclusions	No data were excluded when available.
Replication	The OLINK experiments to confirm elevated plasma GDNF post SARS-CoV-2 infection (Fig 4c) was successfully replicated (Fig .4e).<br>For all other experiments where applicable, all technical replicates were done in duplicates or triplicates. Data were consistent between technical replicates.
Randomization	Animals were randomly assigned to each group with the following exception and explanation. There is an overabundance of females in the study based on the limited availability of animals that we can assign. This is especially true for AGMs, which we do not breed and where we only had 2 males. For these reasons we assigned one male to each group (vaccinated or unvaccinated). We also had two elderly animals, and each group was assigned one older animal. All other animal assignments were randomized.
Blinding	All samples/group assignments were blinded during data acquisition. Blinding was used for pathological assessments and expression levels/

intensity of tissue sections for antigens (SARS-COV-2 proteins), RNA (RNA scope), and glycogen. Computerized non-biased quantitation was performed using HALO (Indica Labs, v3.4.2986.151), which precludes the need for randomization during the analysis.

Reporting for specific materials, systems and methods

We require information from authors about some types of materials, experimental systems and methods used in many studies. Here, indicate whether each material, system or method listed is relevant to your study. If you are not sure if a list item applies to your research, read the appropriate section before selecting a response.

Materials & experimental systems

n/a	Involved in the study
☐	☒ Antibodies
☒	☐ Eukaryotic cell lines
☒	☐ Palaeontology and archaeology
☐	☒ Animals and other organisms
☒	☐ Clinical data
☒	☐ Dual use research of concern
☒	☐ Plants

Methods

n/a	Involved in the study
☒	☐ ChIP-seq
☐	☒ Flow cytometry
☒	☐ MRI-based neuroimaging

Antibodies

Antibodies used

Antibodies were obtained from BD Biosciences (San Jose, California, USA) or BioLegend: CD45-PerCP (BD, clone D058-1283, Cat no. 558411), CD3- BV650 (BD, clone SP34-2, Cat no. 563916), CD4- BV786 (BD, clone L200, Cat no. 563914), CD8- BUV737 (BD, clone RPA-T8, Cat no. 749367), CD28- BV605 (clone CD28.2, Cat no. 562976), CD95- BV711 (clone DX2, Cat no. 563132), CD69- PE-CF594 (clone FN50, Cat no. 562617) and CD107a- BUV395 (BD, clone H4A3, Cat no. 565113). Except for CD45-PerCP (NHP), all antibodies have verified reactivity to humans, and cross reactivity with NHPs. Cells were fixed and permeabilized using BD Fixation/ Permeabilization solution (BD Biosciences) and stained for intracellular cytokines using the following antibodies from BD Biosciences: IL-2- BB700 (clone MQ1-17H12, Cat no. 566405), IL-4- BV421 (clone 8D4-8, Cat no. 562986), Biolegend: TNF- APC (clone MAb1, Cat no. 502912), IFNy- PE-Cy7 (clone 4S.B3, Cat no. 502528), IL-17A- PE (clone BL168, Cat no. 512306).

Validation

The following antibodies from BD have been verified by the company to be reactive towards Non-human primates: CD45-PerCP (BD, clone D058-1283, Cat no. 558411), CD3- BV650 (BD, clone SP34-2, Cat no. 563916), CD4- BV786 (BD, clone L200, Cat no. 563914), CD8- BUV737 (BD, clone RPA-T8, Cat no. 749367), CD28- BV605 (clone CD28.2, Cat no. 562976), CD95- BV711 (clone DX2, Cat no. 563132), CD69- PE-CF594 (clone FN50, Cat no. 562617) and CD107a- BUV395 (BD, clone H4A3, Cat no. 565113), IL-2- BB700 (BD, clone MQ1-17H12, Cat no. 566405). The others relevant to the presented data have been validated by the Flow cytometry core (TNPRC) (Figure 5, Figure S5). All NHP reagent validations can be found in this website https://www.nhpreagents.org/ReactivityDatabase.

Animals and other research organisms

Policy information about studies involving animals; ARRIVE guidelines recommended for reporting animal research, and Sex and Gender in Research

Laboratory animals

African green monkeys (Chlorocebus aethiops sabaeus) (AGMs).
Animal ID Species Sex Age
NC12 AGM Female 7.92
NB94 AGM Female 8.97
NB95 AGM Female 8.97
NB89 AGM Female 8.97
NB88 AGM Female 8.97
NB93 AGM Female 8.97
NC13 AGM Female 7.92
NC11 AGM Female 7.92
NB99 AGM Female 8.97
NB87 AGM Female 8.97
NC18 AGM Female 7.92
NC36 AGM Female 17.92
PB24 AGM Female 19.32
NB85 AGM Male 8.97
NC02 AGM Male 8.97

Wild animals

No wild animals were used in the study. The animals were purchased from Bioqual

Reporting on sex	Of the 15 animals 13 were females, and 2 were males.
Field-collected samples	No field collected samples were used in the stud
Ethics oversight	This study was reviewed and approved by the institutional Animal Care and Use Committee of Tulane University. Animals were cared for in accordance with the NIH's Guide for the Care and Use of Laboratory Animals. Procedures for handling and BSL2, and BSL3 containment of animals were approved by the Tulane University Institutional Biosafety Committee.

Note that full information on the approval of the study protocol must also be provided in the manuscript.

Plants

Seed stocks	N/A
Novel plant genotypes	N/A
Authentication	N/A

Flow Cytometry

Plots

Confirm that:

- [ ] The axis labels state the marker and fluorochrome used (e.g. CD4-FITC).
- [ ] The axis scales are clearly visible. Include numbers along axes only for bottom left plot of group (a 'group' is an analysis of identical markers).
- [x] All plots are contour plots with outliers or pseudocolor plots.
- [ ] A numerical value for number of cells or percentage (with statistics) is provided.

Methodology

Sample preparation	PBMCs were obtained from EDTA blood from African green monkeys. PBMCs were stained with Zombie Aqua (Biolegend, San Diego, CA) for live/dead cell gating, and surface stained using the following pre-titrated antibodies from BD Biosciences (San Jose, California, USA) or BioLegend: CD45-PerCP (BD, clone D058-1283, Cat no. 558411), CD3- BV650 (BD, clone SP34-2, Cat no. 563916), CD4- BV786 (BD, clone L200, Cat no. 563914), CD8- BUV737 (BD, clone RPA-T8, Cat no. 749367), CD28- BV605 (clone CD28.2, Cat no. 562976), CD95- BV711 (clone DX2, Cat no. 563132), CD69- PE-CF594 (clone FN50, Cat no. 562617) and CD107a- BUV395 (BD, clone H4A3, Cat no. 565113). Except for CD45-PerCP (NHP), all antibodies have verified reactivity to humans, and cross reactivity with NHPs. Cells were fixed and permeabilized using BD Fixation/Permeabilization solution (BD Biosciences) and stained for intracellular cytokines using the following antibodies from BBD Biosciences: IL-2- BB700 (clone MQ1-17H12, Cat no. 566405), IL-4- BV421 (clone 8D4-8, Cat no. 562986), Biolegend: TNF- APC (clone MAb1, Cat no. 502912), IFNy- PE-Cy7 (clone 4S.B3, Cat no. 502528), IL-17A- PE (clone BL168, Cat no. 512306). Cells were fixed in 2% PFA and acquired on a BD FACSymphony™ by the Flow cytometry core (TNPRC). Data were analyzed using FlowJo software, version 10.8.1 (Tree Star Inc., Ashland, Oregon, USA) or used for Spectre analysis in R (v4.2.1).
Instrument	BD FACSymphony™
Software	FlowJo software, version 10.8.1
Cell population abundance	The abundance of lymphocyte population was determined based and the forward and side scatter profile.
Gating strategy	We applied standard gating strategy to focus on major subsets within the CD3 T cell population. Of key importance, live cells were gated based on Zombie Aqua/Live/dead staining (Biolegend, San Diego, CA). Singlets were selected based on FSC(A) vs FSC (H). Supplementary Figure 2S shows the gating strategy.

- [x] Tick this box to confirm that a figure exemplifying the gating strategy is provided in the Supplementary Information.
